# Reaction-contingency based bipartite Boolean modelling

**DOI:** 10.1186/1752-0509-7-58

**Published:** 2013-07-08

**Authors:** Max Flöttmann, Falko Krause, Edda Klipp, Marcus Krantz

**Affiliations:** 1 Theoretical Biophysics, Humboldt-Universität zu Berlin, Invalidenstr. 42, Berlin 10115, Germany

**Keywords:** Signal transduction, Systems biology, Boolean modelling, rxncon, Bipartite Boolean

## Abstract

**Background:**

Intracellular signalling systems are highly complex, rendering mathematical modelling of large signalling networks infeasible or impractical. Boolean modelling provides one feasible approach to whole-network modelling, but at the cost of dequantification and decontextualisation of activation. That is, these models cannot distinguish between different downstream roles played by the same component activated in different contexts.

**Results:**

Here, we address this with a bipartite Boolean modelling approach. Briefly, we use a state oriented approach with separate update rules based on reactions and contingencies. This approach retains contextual activation information and distinguishes distinct signals passing through a single component. Furthermore, we integrate this approach in the rxncon framework to support automatic model generation and iterative model definition and validation. We benchmark this method with the previously mapped MAP kinase network in yeast, showing that minor adjustments suffice to produce a functional network description.

**Conclusions:**

Taken together, we (i) present a bipartite Boolean modelling approach that retains contextual activation information, (ii) provide software support for automatic model generation, visualisation and simulation, and (iii) demonstrate its use for iterative model generation and validation.

## Background

Mathematical modelling of large cellular networks is infeasible or impractical, mainly due to the large number of model states and parameters needed to describe these networks. This combinatorial complexity is particularly problematic for signal transduction networks. Their components are often influenced by multiple interaction partners and/or modifications such as phosphorylations, which rapidly combine to a large number of possible configurations – or specific states – of each component. This makes it very difficult to build and parameterise large quantitative models, and computationally very costly to analyse them. However, mathematical analysis of these networks is an important tool for network validation and understanding, urging a development of methods that can be used even for large complex networks.

Boolean modelling provides one of the few feasible approaches to whole-network modelling. While crude, Boolean modelling can prove useful for an initial study of network properties and is often used when quantitative effects do not play a major role in the overall qualitative behaviour of a network. Boolean models were invented for modelling of gene regulatory networks [1], and are now used in a variety of signalling systems [2-4]. Programs supporting Boolean models enable the user to simulate a network, find attractors and perform several analyses on network properties. Although there is software available to “fit” networks to measured data and to translate Boolean models into simple ODE systems [3,5,6], there is no simple software available for the step-by-step analysis and visualisation of Boolean simulations on network graphs with simultaneous state space visualization. Furthermore, the classical Boolean modelling approach [1] does not distinguish between different downstream roles played by a single component activated in different contexts: It only models components (proteins) explicitly, neglecting to differentiate between specific modifications and interactions that provide context specific activity. That is, components are only active or inactive and an activating signal will result in a generic active state. This de-contextualisation of activation makes it impossible to address cross-talk or signal specificity, and makes the classical Boolean approach unsuitable for modelling of interactions between pathways in large complex networks.

Here, we address these shortcomings with a bipartite Boolean modelling approach and supporting software, which integrates model generation, simulation and visualisation. We use a state oriented modelling approach with separate update rules based on reactions and contingencies that corresponds directly to the reaction-contingency (rxncon) format [7]. Briefly, this is a network definition method which separates *reaction* and *contingency* information (reviewed in [8]). The *elemental reactions* and their corresponding *elemental states* define the possible signalling events that can occur and the outcome of these events, respectively. Importantly, different elemental states are not intrinsically mutually exclusive, but instead correspond to sets of specific states sharing a specific property. The *contingencies* define the contextual constraints on these reactions, i.e. which and how elemental states influence downstream elemental reactions. The bipartite Boolean model has the same structure with separate update rules for reactions and for states: States are a function of reactions that produce or consume them, while reactions are functions of states via contingencies. This bipartite Boolean modelling approach retains the contextual information on activation and distinguishes distinct signals passing through the same component. It is implemented and simulated in the classical synchronous Boolean fashion, but retaining the exact network structure of the rxncon input. In this regard, our method goes into a similar direction as the recently published site-specific logical models proposed by [9]. However, it does not require parameterisation whereas the site-specific logical models require threshold parameters on top of a fully parameterised rule based model. The issue of signal specificity in Boolean networks has also been addressed by the recently published mechanistic Boolean approach [10], which relies on specific state based description with the associated scaling issues due to the combinatorial complexity (reviewed in [8]).

We integrate our approach into the rxncon framework to allow automatic model generation, and benchmark the method with the previously mapped MAP kinase network in yeast. Finally, we demonstrate how this modelling approach can be integrated in the network definition process for validation purposes. Taken together, we present a bipartite Boolean modelling approach that retains contextual activation information, can be used without parameterisation, and largely avoids the combinatorial complexity. It also supports automatic model generation from existing network definitions and can therefore be used for iterative network building and validation.

## Results and discussion

### The reaction-contingency information corresponds to a unique Boolean model

**Figure 1:**
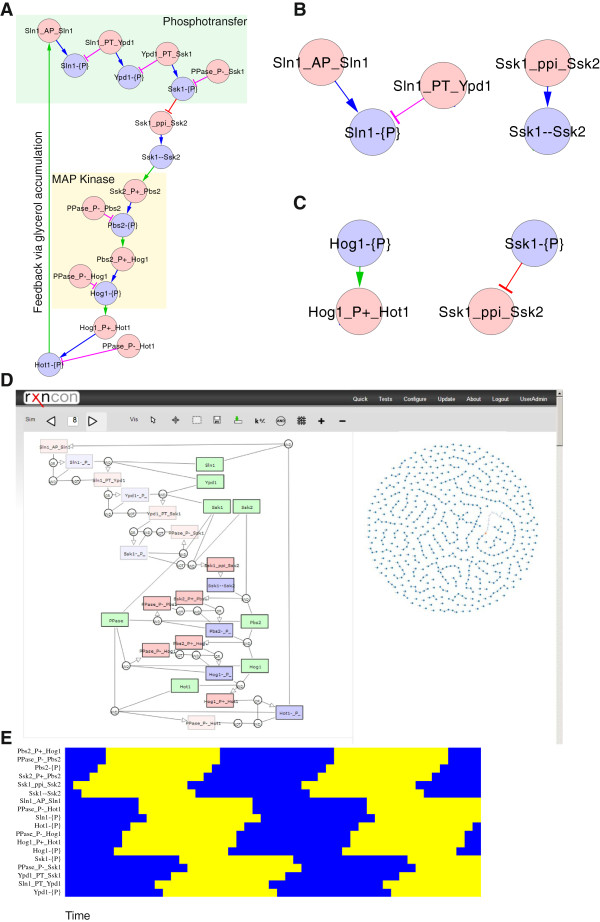
**Generation of a bipartite Boolean model from the rxncon network definition. (A)** The network is defined as *elemental reactions* (red nodes); that produce (blue edges) or consume (purple edges) *elemental states* (blue nodes), and *contingencies* showing how states activate (green edges) or inhibit (red edges) reactions. The elemental reactions correspond to the edges in topological networks, and the contingencies are contextual constraints on the reactions. This simplified version of the high osmolarity glycerol (HOG) pathway contains two modules: The upstream phosphotransfer module (green box) is active when turgor is sufficient, keeping the downstream MAP kinase module (yellow box) inactive. Increased external osmolarity leads to loss of turgor, inactivation of the phosphotransfer module, activation of the MAP kinase module, the output of which again activates the phosphotransfer module (via increased glycerol production and accumulation, leading to turgor recovery, but this part is excluded in this simplified scheme). **(B)** The update rules for states are derived from the reactions as described in the methods. **(C)** The update rules for reactions are derived from the contingencies, and also require that all components taking part in the reaction are present. **(D)** Screenshot from the rxncon Boolean simulation interface with the simplified HOG model. The left side shows the current state of each node, with false nodes appearing faded. Node states can be changed by selecting each node, or by selecting a state in the state plots to the right. The text based network definition (Additional_file_ 1) was pasted into the rxncon quick window and the simulator accessed via the ”Simulate Boolean” button. **(E)** State evolution of the simplified Hog model over two cycles displayed as heat map. Each row corresponds to a single elemental reaction or state, and the colours indicate active (Yellow; True) and inactive (Blue; False) nodes at each time step.

We have previously shown that a rxncon network unambiguously defines a model structure and can be exported to SBML (Systems Biology Markup Language), rule based or agent based formats [7]. While these models can be generated automatically, their behaviour relies heavily on parameter values that must be estimated from empirical data. Here, we complement these export options with a new Boolean format that is able to capture the qualitative network behaviour without any further parameterisation. The model structure mirrors the rxncon regulatory graph (Figure 1A; [7]), and update rules are automatically derived as described in the methods section following a set of fixed export rules that define the Boolean update functions. The bipartite Boolean model is based on two sets of nodes with distinct update rules: Reactions produce or consume states (Figure 1B), and states determine if reactions are active via contingencies (Figure 1C). This bipartite model structure, while not always necessary, has the advantage of simplifying data management for the Boolean model. The model generation requires no further input and hence the rxncon information corresponds to a unique Boolean model.

### Comparison to previous approaches

**Figure 2:**
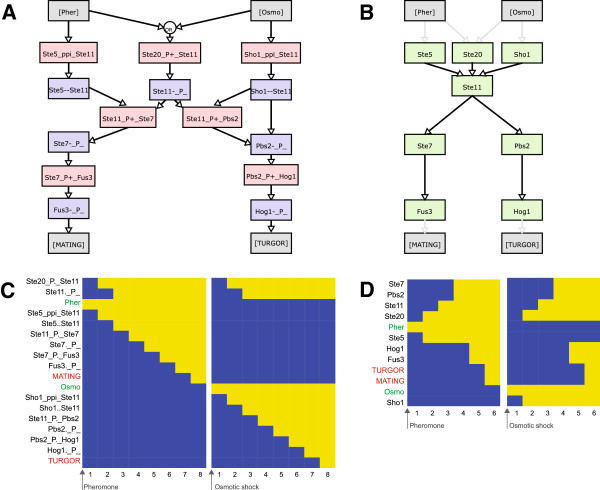
**Comparison of the bipartite and the classical Boolean approaches.** A simplified model of the Pheromone response (MAT) and High osmolarity (HOG) pathways, which share two common kinases (Ste20 and Ste11). This toy model consists of two parallel pathways: the MAT pathway is triggered by a pheromone signal ([Pher]) and initiates mating ([MATING]), and the HOG pathway is induced by hyperosmotic stress ([Osmo]) and triggers turgor recovery ([TURGOR]). The network structure of **(A)** the bipartite Boolean model and **(B)** a classical Boolean model have topologies corresponding to the regulatory graph **(A)** and reaction graph **(B)**, respectively, of the same rxncon network definition (Additional file 2: Table S2). The bipartite Boolean code was automatically generated as described in the Methods section, while the classical Boolean code was created manually based on the reaction graph topology. **(C, D)** State evolution of the MAPK network displayed as heat map. Yellow and blue indicate active (True) and inactive (False) nodes, respectively. **(C)** The bipartite Boolean model can distinguish different input signals and activate only their specific outputs accordingly. **(D)** The network structure of a classical approach is simpler, as it does not consider states and reactions as separate from components, but it is not able to maintain signal specificity and always activates both outputs in response to either input signal.

To show the differences between our approach of Boolean model creation and classical Boolean models we used a small example network (Figure 2). The standard translation of a biological process into the Boolean formalism is phenomenological and based on a purely topological map of the system (Figure 2B). It converts the Boolean states of e.g. proteins Ste5 and Ste20 into the Boolean state of protein Ste11 and in turn to downstream proteins. Compared to that, our approach (Figure 2A) is more detailed and includes variables for each protein state and each reaction, i.e. Ste5 binding Ste11, Ste20 phosphorylating Ste11 which act in combination on the downstream signal. Our approach has the advantage of distinguishing between upstream signals that act on one component and translating it into different downstream activations. This higher specificity comes at the price of more variables and a larger state space, and therefore higher complexity. Simulation results of the two models (Figure 2C, D) clearly show the separation of the signals. The bipartite approach (C) leads to the activation of the correct output, while the classic approach (D) always activates both outputs as it is unable to keep the signals separate.

### Integrated model generation, simulation and visualisation

To further facilitate integration of model creation and validation, we extended the rxncon tool to simulate and visualise Boolean models. These functions are accessible directly within the user interface of rxncon; using BooleanNet for simulation [11] and the biographer library for visualisation [12]. The simulation interface visualises the network as an activity flow (AF) diagram according to the Systems Biology Graphical Notation standard (SBGN; [13]). The SBGN-AF representation contains the reactions and states from the rxncon regulatory graph, but also includes the nodes for each of the network components themselves (Figure 1D; left). It comes in two different styles: the default style visualises all influences according to the Boolean update rules, while the alternative style mirrors the regulatory graph format. The regulatory graph is more easily accessible as it leaves out the influence of components on reactions and a large number of Boolean operators. Both styles include all components, reactions, states, inputs and outputs; which can be turned on or off individually to alter the initial state of the simulation. The network layout can be imported from file and/or edited manually. The possible state trajectories are calculated automatically and visualised within the simulator (Figure 1D; right). The complete state space can only be calculated and visualised for small models, while for larger models the calculation is limited to states reachable from a limited set of starting states. The state space visualisation allows the user to access a specific state by simply selecting it, and also clearly identifies point and cyclic attractors. The modelling interface includes layout algorithms and the node positions can be saved to let previously existing nodes retain their positions as new nodes are added. Hence, this extension provides support for iterative model generation, visualisation and simulation; facilitating integration of the three steps in the network reconstruction process. As we show below, the bipartite Boolean simulation provides a powerful albeit qualitative validation tool. The iteration between model creation and qualitative model validation provides for quality assurance in the model creation process without the need of expensive – if not infeasible – parameterisation and quantitative simulation.

### Iterative model building and validation

**Figure 3:**
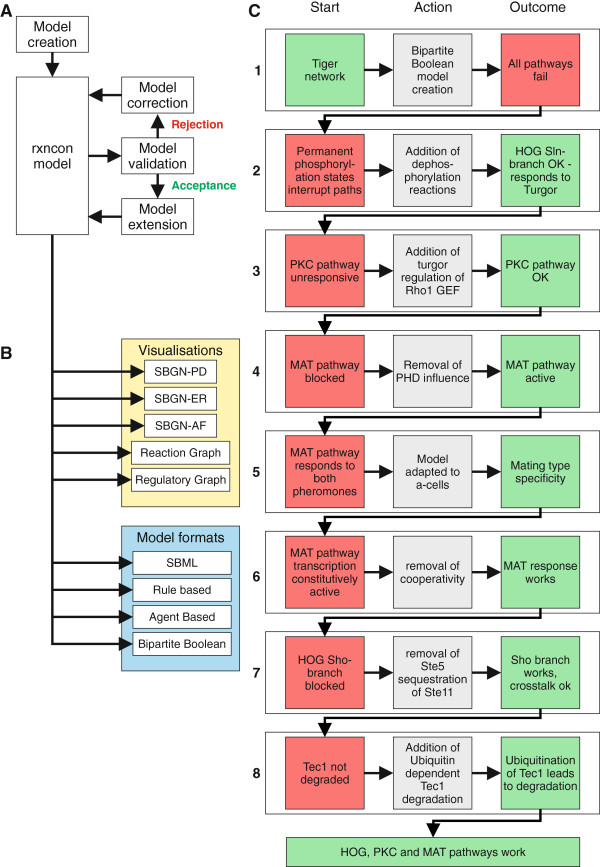
**Iterative model building and validation as a tool to guide and validate network reconstruction. (A)** Idealised workflow for model building: Model extensions and improvements are done in small steps, with each step being evaluated as a Boolean model. **(B)** The rxncon database underlying the Boolean model is fully compatible with the other rxncon features, including a range of visualisations and automatic model generation in formats suitable for quantitative modelling. **(C)** The iterative improvement applied on the yeast MAP kinase network. Only a limited number of changes were needed to make the HOG, PKC and MAT pathways functional (Figure 5), most of which are in line with existing knowledge as discussed in the main text. The single largest change was the addition of 50 hypothetical dephosphorylation reactions (Additional file 3: Table S3).

The potentially most potent contribution of the integration of Boolean model generation and simulation in the network definition framework is that it enables iterative model building and validation (Figure 3A). The idealised work flow starts from an existing model or a small network reconstruction, which is translated into a bipartite Boolean model and simulated to confirm that the current reconstruction can reproduce the network’s *in vivo* function qualitatively. Ideally, the iteration uses small steps to immediately identify missing and/or erroneous features and to constantly keep the model consistent with *in vivo* observations. This can be done without any overhead due to Boolean model creation, as the network definition format is identical to that used in all other rxncon features (Figure 3B). The input used to create the bipartite Boolean model can also be exported to the standard SBML format or to formats for rule or agent based modelling; as well as to a range of visual formats, including the SBGN formats. Hence, the Boolean analysis can easily be integrated as a validation step in a modelling effort aiming for a quantitative model without duplication of work.

### Validation and extension of the yeast MAP kinase network

**Figure 4:**
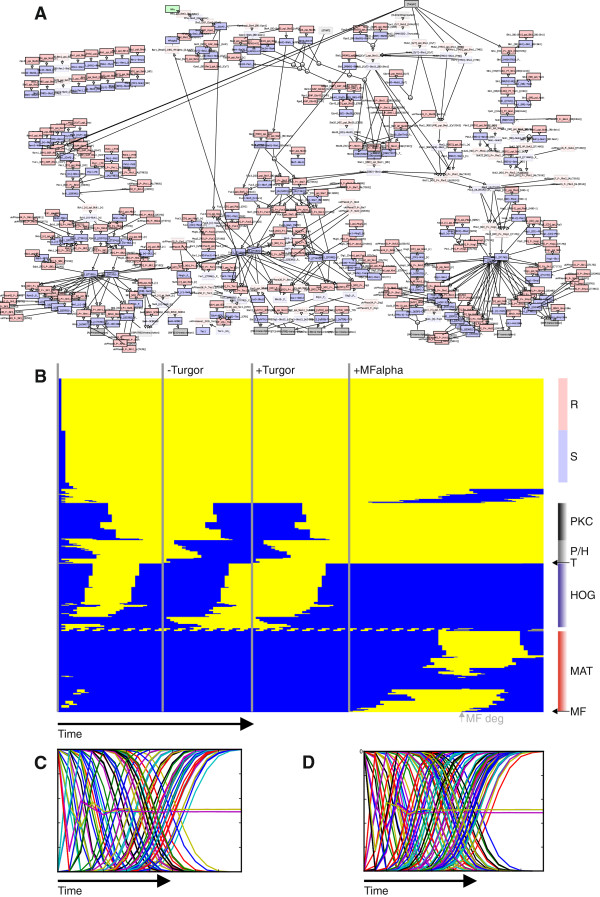
**Simulation of the complete MAP kinase network within the rxncon Boolean simulator. (A)** Snapshot of the simulation. The network was generated from Additional file 4: Table S4 and the layout was imported from Additional file 5. **(B)** State evolution of the MAPK network displayed as heat map. Yellow and blue indicate active (True) and inactive (False) nodes, respectively. Grey vertical lines indicate simulation start and perturbations after the system reaches its steady states. Simulation starts using the default settings (+Turgor, -MFalpha, -Ste3, +Tec1; Figure 5). When it reaches the steady state, turgor (T) is turned off (t=27), then switched on again (t=50) and finally we add MFalpha (MF; t=75), which is degraded as the cells adapt (grey arrow; “MF deg”). The Pathway components cluster together in their state evolution (See Methods), including a group of early PKC pathway components that cluster with the components of the (negative) Sln-branch of the Hog pathway (P/H). Most mating pathway components (MAT) are first activated and then deactivated after their activation lead to mating factor degradation (MF deg; a negative feedback). The unregulated reactions (R) and states (S) are turned on at time step 1 and 2, respectively, and stay constitutively active. **(C, D)** Asynchronous simulation of the network from the default steady state (t=25 in B). The lineplots show the average of 1000 simulations for each variable that change during the simulation: **(C)** MFalpha stimulation and **(D)** Osmotic shock. Nearly all variables reach the same states in each of the simulation. The only exceptions are four connected variables that form a small negative feedback cycle that constitute a small cyclic attractor in the synchronous updating, which ends in a randomly chosen point attractor in each of the asynchronous simulations (one pair on, the other pair off).

We revisited the carefully curated MAP kinase network of baker’s yeast, *Saccharomyces cerevisiae*[7], henceforth referred to as Tiger network. This is a literature based network reconstruction that was performed in a controlled vocabulary supporting automatic model generation, but the network was never computationally analysed: The bipartite Boolean model analysis presented here is the first analysis of a model derived from this network reconstruction. The simulation enabled us to test whether the information in the network reconstruction is sufficient to recreate the expected behaviour reflecting the existing biological knowledge about the system or not. The MAPK network controls cell morphology, mating and mitosis in response to environmental perturbations and hormones. The high osmolarity glycerol (HOG) pathway responds to increased extracellular osmolarity and turgor loss via two branches converging on the MAP kinase kinase Pbs2 (reviewed in [14]). It is antagonistic to the protein kinase C (PKC) pathway, which among other stimuli responds to increased turgor (reviewed in [15]). The mating (MAT) pathway is active in haploids, in which mating type specific receptors respond to pheromones from cells with the complementary mating type (reviewed in [16]). Less well characterised, the pseudohyphal differentiation (PHD) pathway is thought to regulate growth pattern in response to nutrient depletion. To assess the accuracy and completeness of this network curation, we generated the corresponding bipartite Boolean model to determine which additional features would be needed to (qualitatively) capture the physiological behaviour of the network (Figure 4A).

**Figure 5:**
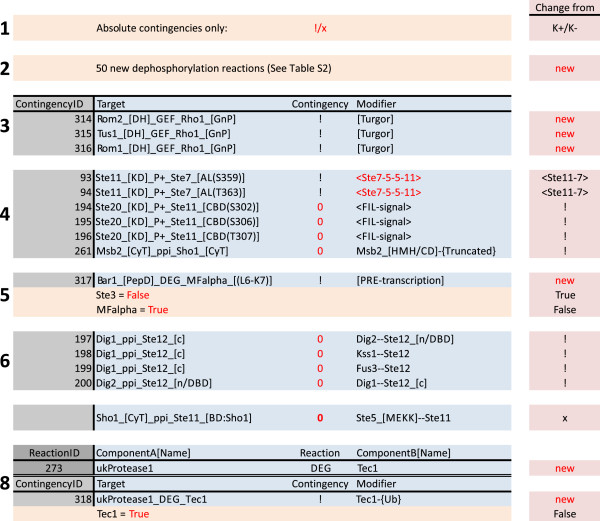
**Improvements to the MAP kinase model.** The complete list of the improvements needed to generate a functional MAP kinase network (Additional file 4: Table S4). Block numbers indicate the step according to Figure 3C. Step 1 is the translation of the Tiger network to a qualitative model, i.e. changing all quantitative contingencies; “K+” and “K-”, to qualitative contingencies; “!” and “x”. The remaining changes either added reactions (step 2 and 8) or contingencies (step 3, 5 and 8), or altered contingencies (step 4, 6, and 7) or starting states (step 5 and 8).

The network was translated into a bipartite Boolean model assuming all contingencies were absolute, as Boolean simulations cannot deal with quantitative modifiers (Figure 3C; Figure 5). Not surprisingly, we found that this network definition is insufficient to predict the network behaviour and proceeded to identify the missing features. Most importantly, the Tiger network contains 50 phosphorylation reactions that lack a corresponding dephosphorylation reaction. To address this, we added 50 hypothetical dephosphorylation reactions to make all phosphorylation states reversible (Additional file 3: Table S3). Phosphorylation reactions are generally reversible, and the lack of the corresponding dephosphorylation reactions in the Tiger network most likely corresponds to a gap in our knowledge rather than their absence *in vivo*. Adding these highly plausible reactions without any assumptions on their regulation was enough to make the Sln1 branch and hence the Hog pathway functional, as measured by its ability to respond to turgor.

Next, we turned our attention to the PKC pathway. It has been reported to respond to increasing osmolarity [17], although the sensing mechanism remains unclear. To make it turgor sensitive, we simply added a turgor requirement for the guanine nucleotide exchange (GEF) of Rho1. While mechanistically unsatisfactory, this is sufficient to make the PKC pathway responsive to turgor. Importantly, no additional modifications are needed downstream for the signal to reach its targets.

The MAT pathway required more complex adjustments, in part due to the interconnection with the HOG and PHD pathways. Yeast mating only occurs between haploid yeast cells of complementary mating types; MATa and MATalpha. To simulate the well studied MATa-cells, we removed the MATalpha-cell specific mating receptor (Ste3), and added a negative feedback loop on the pheromone response by allowing degradation of alpha factor only after gene induction of Bar1. Next, we eliminated the interference from the only partially defined PHD pathway. In the Tiger network, the PHD and MAT pathway stimulates some of the same components, which was translated as absolute requirements hence blocking these reactions completely in the Boolean model. To remove this block, we simply removed the influence of the PHD pathway by removing the effect of four contingencies, and corrected the requirement for two others (Figure 5). Finally, we removed the cooperative binding of the downstream transcription factors (which again were interpreted as absolute requirements and hence blocking reactions unduly), and added the ubiquitination dependent degradation of the Tec1 transcription factor, which was missing in the Tiger network. In total, we needed to adjust only ten out of 281 contingencies, and add one reaction and one contingency to make our Boolean model of the MAT pathway work according to our current understanding.

We resolved the HOG-MAT crosstalk by removing one final contingency, namely the ability of Ste5 recruitment of Ste11 to block the interaction of Ste11 and Sho1. While this block is likely true for each Ste11 bound to Ste5, the amount of Ste11 in the cell vastly exceeds that of Ste5, making a complete inhibition by stoichiometric binding impossible [18].

Taken together, the main changes were addition of 50 new dephosphorylation reactions and turgor regulation of Rho1. The dephosphorylation reactions, at least, are likely to exist *in vivo* and reflect a clear bias in experimental evidence towards characterisation of kinases. Additionally, we corrected the assumption of absolute effects of 4% of all contingencies, which is a surprisingly low number considering the strength of the assumptions that all quantitative regulatory effects can be considered to be functionally absolute requirements. We also added transcriptional induction of Bar1 [19] and Tec1 degradation after ubiquitination [20]. Hence, apart from the dephosphorylation reactions and the turgor regulation of Rho1, the changes are either a relaxation of the assumption that all regulatory effects can be described as absolute, or based on empirical evidence; and overall very few. This shows that we are close to a functional understanding of the HOG, PKC and MAT pathways; that this functional understanding can be expressed within the rxncon formalism; and that the iterative model building and bipartite Boolean simulation is a potent tool to identify and correct missing or erroneous features in even very large models.

## Conclusions

We present a bipartite Boolean modelling approach supported by automatic model generation, simulation and visualisation in the rxncon software. Our Boolean approach retains contextual activation information and avoids inappropriate pathway crosstalk, even when the signal passes through shared components. The Boolean export and simulation complement the existing rxncon exports to SBML, rule based and agent-based models, and graphical formats such as the SBGN formats. Furthermore, we demonstrate the use of Boolean modelling for model validation and show how it can be integrated in the model construction process. The simple Boolean model creation without further necessary information sets our approach apart from similar methods proposed before. We envisage this iterative process of model building and qualitative validation to be a useful tool in construction of network maps and even quantitative mathematical models.

## Methods

### Software

The rxncon tool is released under the GNU Lesser General Public License (LGPL) open source license, and can be freely downloaded from http://rxncon.org. The Boolean Simulation can also be done online, without installation. It is based on the web framework web2py (http://www.web2py.com). The Boolean simulation uses BooleanNet (https://code.google.com/p/booleannet), the simulation is visualised using the Biographer software (http://biographer.biologie.hu-berlin.de), and the state spaces are displayed using the d3 javascript library (http://d3js.org/). The required libraries are included in the distribution packages and do not need to be installed separately.

### Model generation

The Boolean model structure directly corresponds to the rxncon regulatory graph [7]. This bipartite graph has elemental reactions and states as nodes, reaction effects as reaction-to-state edges, and contingencies as state-to-reaction edges. Our approach of encoding the reaction information into Boolean logic uses the same bipartite partitioning and has separate update functions for the reactions, states, and input and output nodes. To be able to use a standard translation from the rxncon format to the Boolean format, we had to make certain assumptions about the dependencies that are described in the following.

In our Boolean models, reactions depend on the states that are given as their contingencies and the components that are involved. Contingencies giving quantitative and absolute requirements (k+/!) as well as components go into the equation with an AND operator. States given in negative contingencies (k-/x) simply are negated with a NOT operator. Components are part of the Boolean model, but are not influenced by any other components and are therefore considered constant. Boolean nodes defined in the rxncon format are flattened in the update function of the reactions in the Boolean format by adding them recursively to the function. Reaction updates are illustrated by the phosphorylation of Hot1 (a transcription factor that is activated by the HOG pathway) by Hog1 (Hog1_P + _Hot1), which requires that Hog1 is phosphorylated (Hog1-{P}; Figure 1 C). The reaction Hog1_P + _Hot1 is true if Hog1 is true AND Hot1 is true AND Hog1-{P} is true: Hog1 _ P + _ Hot1(t + 1)  =  Hog1(t) ∧ Hot1(t) ∧ Hog1 ‒ {P} (t). Protein-protein interaction between Ssk1 and Ssk2 (Ssk1_ppi_Ssk2) is inhibited by Ssk1 phosphorylation (Ssk1-{P}). This yields: Ssk1 _ ppi _ Ssk2 (t + 1)  =  Ssk1(t)  ∧  Ssk2(t)  ∧ *¬*Ssk1 ‒ {P}(t).

Update functions of states are built up from the producing reactions, the consuming reactions, the involved components, and the state itself. Components are absolute requirements for the state to be true, while the exact structure of the update function depends on the reaction types the state is involved in. Reversible production reactions need to be set to true to keep the state active, because reveresible reactions are considered to decay their states when set to false. In contrast, irreversible reactions cannot switch produced states to false. Output nodes are treated in the same way as states, while input nodes are constantly either true or false.

Updating states can be exemplified by the reactions depicted in Figure 1B. The state Sln1-{P} of protein Sln1 is produced by auto-phosphorylation and consumed by phosphotransfer to Ypd1. This would be updated by the following rule: Sln1–{P} (t + 1) =  Sln1 _ AP _ Sln1(t)  ∨  Sln1–{P}(t)  ∧ *¬*Sln1 _ PT _ Ypd1(t). Once the state is true, it cannot be set to false by the producing reaction anymore, because the reaction is irreversible. A different example is the Ssk1-Ssk2 dimer (Ssk1--Ssk2) that is produced by the protein-protein interaction between Ssk1 and Ssk2 (Ssk1_ppi_Ssk2). It follows the update rule: Ssk1--Ssk2(t + 1) = Ssk1 _ ppi _ Ssk2(t). The state would decay if the reaction was false, as protein-protein interactions (ppi:s) are defined as a reversible reaction. For a more comprehensive example please refer to Additional file 1: Table S1 and the included version of the Tiger network.

Additionally, we simulated the working model with asynchronous updating. Due to the rather linear nature of the network and the lack of negative feedbacks we don’t see large differences in the attractor landscape (Figure 4C, D) reachable from the simulated states.

### Visualization

The time course of the full MAP kinase model in Figure 4B was generated using BooleanNet and visualized by the Heatplus package in R. This was done by clustering the model entities according to their states over time and displaying states in a heatmap. States that do not change over the whole time course were left out.

### Creation of the rxncon input file

The input file can be created as an Excel file (recommended; template provided with the rxncon software) or as text based direct input (described further below). The Excel input consists of two lists; the reaction list and the contingency list. The reaction list defines the network topology. Each reaction is defined by two components and a relationship (reaction) between them. In the minimal format as used for the example network in Figure 1, only reaction and component names are required (columns L, P and Q in sheet “(I) Reaction list”). Reaction and state IDs are automatically generated in the grey columns (B-F). Importantly; the components are always entered in their basic state even when previous modifications are required. These requirements are defined in the contingency list. Each constraints on a reaction must be defined as a contingency, and each contingency consist of three parts: A target (column B), which identifies the reaction that is affected; a contingency (column C), which defines how the target reaction is affected; and a modifier (column D), which identifies the state causing the effect. The reactions must correspond to the reaction IDs in column B of sheet “(I) Reaction list”, and the states to the state IDs in column C and/or D in the same sheet. The easiest method to add contingency information is to link the target cells to reaction cells and effector cells to state cells (both in sheet: “(I) Reaction list”). This also ensures consistency if the reaction sheet is updated. More complex models may make use of Boolean statements, inputs and outputs, as described further on http://rxncon.org and in Tiger et al. [7]. The excel file is loaded directly into the rxncon tool from which all export functions as well as the simulation interface will be available.

Alternatively, a model can be defined directly as text input. Reactions need to be written exactly as they would appear in column B of sheet “(I) Reaction list” (see http://rxncon.org/test for examples). Contingencies would be added directly to each reaction after , as shown in the more complex examples on the same page. The text based definition is written or pasted into the “quick” user interface view, from which a subset of the rxncon functions are available. However, the direct text format does not have the database features of the Excel format, which facilitate reusability, documentation and links to references.

## Competing interests

The authors declare that they have no competing interests.

## Authors’ contribution

MF and MK designed the bipartite Boolean modelling framework. MF and FK implemented the software. FK integrated the software with the rxncon framework. MK adapted the MAPK network and drafted the manuscript. MK and EK contributed biological and theoretical background knowledge, respectively. EK provided the research environments and contributed to completion of the manuscript. All authors read, edited and approved the final manuscript.

## Supplementary Material

Additional file 1: Table S1 The simplified Hog model. (A) Input string for the quick generation of the simplified HOG model used in Figure 1. (B) Boolean functions for the update of states and (C) reactions for the corresponding bipartite Boolean model. Click here for file

Additional file 2: Table S2 The cross-talk example. (A) Input string for the quick generation of the cross talk example used in Figure 2. (B, C) The simulation code for (B) the bipartite Boolean model and (C) the classical Boolean model. Click here for file

Additional file 3: Table S3 Dephosphorylation reactions added to the MAP kinase network. 50 reactions were added to the MAP kinase network definition to make phosphorylation states reversible. Each of these reactions was assigned to an unknown phosphatase (ukPPase). Click here for file

Additional file 4: Table S4 The resulting MAP kinase model. The complete rxncon MAP kinase model after the changes listed in Figure 5. This model can be imported into rxncon to generate the Boolean model or other visualisation and/or models. Note that the model remains qualitative only (no K+/K-). The network layout can be imported from Additional file 5. Click here for file

Additional file 5 Contains the layout coordinates for the original MAP kinase network from Tiger et al. used to import layout to the rxncon simulation interface. Click here for file
