# Supplementary material for: Reaction-contingency based bipartite Boolean modelling
Source: BMC Syst Biol. 2013 Jul 8;7:58. doi: 10.1186/1752-0509-7-58 (PMC3710479; doi:10.1186/1752-0509-7-58)
Supplement: Additional file 1: Table S1 — The simplified Hog model. (A) Input string for the quick generation of the simplified HOG model used in Figure 1. (B) Boolean functions for the update of states and (C) reactions for the corresponding bipartite Boolean model. [file 1752-0509-7-58-S1.pdf]

	ContingencyID	Target	Contingency	Modifier	Change from
1		Absolute contingencies only:	!/x		K+/K-
2		50 new dephosphorylation reactions (See Table S2)			new
3	314	Rom2_[DH]_GEF_Rho1_[GnP]	!	[Turgor]	new
	315	Tus1_[DH]_GEF_Rho1_[GnP]	!	[Turgor]	new
	316	Rom1_[DH]_GEF_Rho1_[GnP]	!	[Turgor]	new
4	93	Ste11_[KD]_P+_Ste7_[AL(S359)]	!	<Ste7-5-5-11>	<Ste11-7>
	94	Ste11_[KD]_P+_Ste7_[AL(T363)]	!	<Ste7-5-5-11>	<Ste11-7>
	194	Ste20_[KD]_P+_Ste11_[CBD(S302)]	0	<FIL-signal>	!
	195	Ste20_[KD]_P+_Ste11_[CBD(S306)]	0	<FIL-signal>	!
	196	Ste20_[KD]_P+_Ste11_[CBD(T307)]	0	<FIL-signal>	!
	261	Msb2_[CyT]_ppi_Sho1_[CyT]	0	Msb2_[HMH/CD]-{Truncated}	!
5	317	Bar1_[PepD]_DEG_MFalpha_[(L6-K7)]	!	[PRE-transcription]	new
		Ste3 = False			True
		MFalpha = True			False
6	197	Dig1_ppi_Ste12_[c]	0	Dig2--Ste12_[n/DBD]	!
	198	Dig1_ppi_Ste12_[c]	0	Kss1--Ste12	!
	199	Dig1_ppi_Ste12_[c]	0	Fus3--Ste12	!
	200	Dig2_ppi_Ste12_[n/DBD]	0	Dig1--Ste12_[c]	!
		Sho1_[CyT]_ppi_Ste11_[BD:Sho1]	**0**	Ste5_[MEKK]--Ste11	x
8	ReactionID	ComponentA[Name]	Reaction	ComponentB[Name]	
	273	ukProtease1	DEG	Tec1	new
	ContingencyID	Target	Contingency	Modifier	
	318	ukProtease1_DEG_Tec1	!	Tec1-{Ub}	new
		Tec1 = True			False

Table 1
