# Supplementary material for: Reaction-contingency based bipartite Boolean modelling
Source: BMC Syst Biol. 2013 Jul 8;7:58. doi: 10.1186/1752-0509-7-58 (PMC3710479; doi:10.1186/1752-0509-7-58)
Supplement: Additional file 2: Table S2 — The cross-talk example. (A) Input string for the quick generation of the cross talk example used in Figure 2. (B, C) The simulation code for (B) the bipartite Boolean model and (C) the classical Boolean model. [file 1752-0509-7-58-S2.pdf]

A

Sln1_AP_Sln1; ! Hot1-{P}
Sln1_PT_Ypd1
Ypd1_PT_Ssk1
Ssk1_ppi_Ssk2; x Ssk1-{P}
Ssk2_P+_Pbs2; ! Ssk1--Ssk2
Pbs2_P+_Hog1; ! Pbs2-{P}
Hog1_P+_Hot1; ! Hog1-{P}
PPase_P-_Ssk1
PPase_P-_Pbs2
PPase_P-_Hog1
PPase_P-_Hot1

B

$$\text{Sln1-\{P\}} = \text{Sln1\_AP\_Sln1} \lor \neg\text{Sln1\_PT\_Ypd1} \land \text{Sln1-\{P\}}$$
$$\text{Ssk1-\{P\}} = \text{Ypd1\_PT\_Ssk1} \lor \neg\text{PPase\_P-\_Ssk1} \land \text{Ssk1-\{P\}}$$
$$\text{Hot1-\{P\}} = \text{Hog1\_P+\_Hot1} \lor \neg\text{PPase\_P-\_Hot1} \land \text{Hot1-\{P\}}$$
$$\text{Hog1-\{P\}} = \text{Pbs2\_P+\_Hog1} \lor \neg\text{PPase\_P-\_Hog1} \land \text{Hog1-\{P\}}$$
$$\text{Ypd1-\{P\}} = \text{Sln1\_PT\_Ypd1} \lor \neg\text{Ypd1\_PT\_Ssk1} \land \text{Ypd1-\{P\}}$$
$$\text{Pbs2-\{P\}} = \text{Ssk2\_P+\_Pbs2} \lor \neg\text{PPase\_P-\_Pbs2} \land \text{Pbs2-\{P\}}$$
$$\text{Ssk1--Ssk2} = \text{Ssk1\_ppi\_Ssk2}$$

C

$$\text{Hog1\_P+\_Hot1} = Hog1 \land Hot1 \land \text{Hog1-\{P\}}$$
$$\text{Ssk1\_ppi\_Ssk2} = Ssk1 \land Ssk2 \land \neg\text{Ssk1-\{P\}}$$
$$\text{PPase\_P-\_Hot1} = PPase \land Hot1 \land \text{Hot1-\{P\}}$$
$$\text{PPase\_P-\_Pbs2} = PPase \land Pbs2 \land \text{Pbs2-\{P\}}$$
$$\text{PPase\_P-\_Ssk1} = PPase \land Ssk1 \land \text{Ssk1-\{P\}}$$
$$\text{PPase\_P-\_Hog1} = PPase \land Hog1 \land \text{Hog1-\{P\}}$$
$$\text{Ypd1\_PT\_Ssk1} = Ypd1 \land Ssk1 \land \text{Ypd1-\{P\}}$$
$$\text{Pbs2\_P+\_Hog1} = Pbs2 \land Hog1 \land \text{Pbs2-\{P\}}$$
$$\text{Sln1\_PT\_Ypd1} = Sln1 \land Ypd1 \land \text{Sln1-\{P\}}$$
$$\text{Ssk2\_P+\_Pbs2} = Ssk2 \land Pbs2 \land \text{Ssk1--Ssk2}$$
$$\text{Sln1\_AP\_Sln1} = Sln1 \land Sln1 \land \text{Hot1-\{P\}}$$

Table S1
