# Supplementary material for: Reaction-contingency based bipartite Boolean modelling
Source: BMC Syst Biol. 2013 Jul 8;7:58. doi: 10.1186/1752-0509-7-58 (PMC3710479; doi:10.1186/1752-0509-7-58)
Supplement: Additional file 3: Table S3 — Dephosphorylation reactions added to the MAP kinase network. 50 reactions were added to the MAP kinase network definition to make phosphorylation states reversible. Each of these reactions was assigned to an unknown phosphatase (ukPPase). [file 1752-0509-7-58-S3.pdf]

A

```
Ste20_P+_Ste11; ! <Input>
<Input>; OR [Pher]; OR [Osmo]
Ste5_ppi_Ste11; ! [Pher]
Sho1_ppi_Ste11; ! [Osmo]
Ste11_P+_Ste7; ! Ste11-{P}; ! Ste5--Ste11
Ste11_P+_Pbs2; ! Ste11-{P}; ! Sho1--Ste11
Ste7_P+_Fus3; ! Ste7-{P}
Pbs2_P+_Hog1; ! Pbs2-{P}
[MATING]; ! Fus3-{P}
[TURGOR]; ! Hog1-{P}
```

B

```
Sho1 = True
Fus3 = True
Ste20 = True
Ste11 = True
Hog1 = True
Ste7 = True
Ste5 = True
Pbs2 = True

Ste11-_P_ = False
Sho1--Ste11 = False
Ste5--Ste11 = False
Hog1-_P_ = False
Fus3-_P_ = False
Ste7-_P_ = False
Pbs2-_P_ = False

Ste7_P+_Fus3 = False
Ste5_ppi_Ste11 = False
Ste11_P+_Ste7 = False
Ste11_P+_Pbs2 = False
Sho1_ppi_Ste11 = False
Pbs2_P+_Hog1 = False
Ste20_P+_Ste11 = False

Pher = False
MATING = False
TURGOR = False
Osmo = False

MATING *= Fus3-_P_
TURGOR *= Hog1-_P_

Ste11-_P_ *= (Ste20_P+_Ste11) or Ste11-_P_
Sho1--Ste11 *= (Sho1_ppi_Ste11)
Ste5--Ste11 *= (Ste5_ppi_Ste11)
Hog1-_P_ *= (Pbs2_P+_Hog1) or Hog1-_P_
Fus3-_P_ *= (Ste7_P+_Fus3) or Fus3-_P_
Ste7-_P_ *= (Ste11_P+_Ste7) or Ste7-_P_
Pbs2-_P_ *= (Ste11_P+_Pbs2) or Pbs2-_P_

Ste7_P+_Fus3 *= Ste7 and Fus3 and Ste7-_P_
Ste5_ppi_Ste11 *= Ste5 and Ste11 and Pher
Ste11_P+_Ste7 *= Ste11 and Ste7 and Ste11-_P_ and Ste5--Ste11
Ste11_P+_Pbs2 *= Ste11 and Pbs2 and Ste11-_P_ and Sho1--Ste11
Sho1_ppi_Ste11 *= Sho1 and Ste11 and Osmo
Pbs2_P+_Hog1 *= Pbs2 and Hog1 and Pbs2-_P_
Ste20_P+_Ste11 *= Ste20 and Ste11 and (Pher or Osmo)
```

C

```
Sho1 = False
Fus3 = False
Ste20 = False
Ste11 = False
Hog1 = False
Ste7 = False
Ste5 = False
Pbs2 = False

Pher = False
MATING = False
TURGOR = False
Osmo = False

MATING *= Fus3
TURGOR *= Hog1

Sho1 *= Osmo
Fus3 *= Ste7
Ste20 *= Pher or Osmo
Ste11 *= Ste5 and Ste20 or Sho1 and Ste20
Hog1 *= Pbs2
Ste7 *= Ste11
Ste5 *= Pher
Pbs2 *= Ste11
```

Table S2
