# Supplementary material for: Reaction-contingency based bipartite Boolean modelling
Source: BMC Syst Biol. 2013 Jul 8;7:58. doi: 10.1186/1752-0509-7-58 (PMC3710479; doi:10.1186/1752-0509-7-58)
Supplement: Additional file 4: Table S4 — The resulting MAP kinase model. The complete rxncon MAP kinase model after the changes listed in Figure 5. This model can be imported into rxncon to generate the Boolean model or other visualisation and/or models. Note that the model remains qualitative only (no K+/K-). The network layout can be imported from Additional file 5. [file 1752-0509-7-58-S4.pdf]

L	M	N	O	P-	Q	R	S	T
ComponentA[Name]	ComponentA[Domain]	ComponentA[Subdomain]	ComponentA[Residue]	Reaction	ComponentB[Name]	ComponentB[Domain]	ComponentB[Subdomain]	ComponentB[Residue]
ukPPase1				P-	Sst2			S539
ukPPase2				P-	Ste5			
ukPPase3				P-	Pkc1	AL		T983
ukPPase4				P-	Msg5			
ukPPase5				P-	Rlm1	c		S427
ukPPase6				P-	Rlm1	c		T439
ukPPase7				P-	Sir3			S275
ukPPase8				P-	Swi4			
ukPPase9				P-	Swi6			S238
ukPPase10				P-	Rck2	c		S519
ukPPase11				P-	Sic1			T173
ukPPase12				P-	Sko1	n		S108
ukPPase13				P-	Sko1	n		S113
ukPPase14				P-	Sko1	n		S126
ukPPase15				P-	Rck2	Ser		
ukPPase16				P-	Pbs2	AL		S514
ukPPase17				P-	Pbs2	AL		T518
ukPPase18				P-	Ssk2			T1460
ukPPase19				P-	Ste11	CBD		S302
ukPPase20				P-	Ste11	CBD		S306
ukPPase21				P-	Ste11	CBD		T307
ukPPase22				P-	Ssk1	RR		D544
ukPPase23				P-	Dig1			
ukPPase24				P-	Dig2			
ukPPase25				P-	Far1			T306
ukPPase26				P-	Ste12			
ukPPase27				P-	Ste7			
ukPPase28				P-	Tec1			T273
ukPPase29				P-	Ste7	AL		S359
ukPPase30				P-	Ste7	AL		T363
ukPPase31				P-	Ste20	SerThr		
ukPPase32				P-	Bck1			S939
ukPPase33				P-	Smp1			S348
ukPPase34				P-	Smp1			S357
ukPPase35				P-	Smp1			T365
ukPPase36				P-	Smp1			S376
ukPPase37				P-	Hot1			S30
ukPPase38				P-	Hot1			S70
ukPPase39				P-	Hot1			S153
ukPPase40				P-	Hot1			S360
ukPPase41				P-	Hot1			S410
ukPPase42				P-	Rom2			
ukPPase43				P-	Mkk1			S377
ukPPase44				P-	Mkk1			T381
ukPPase45				P-	Mkk2			S370
ukPPase46				P-	Mkk2			T374
ukPPase47				P-	Sir3			S282
ukPPase48				P-	Sir3			S289
ukPPase49				P-	Sir3			S295
ukPPase50				P-	Tec1			T276

Table S3
